# High-dose maternal folic acid supplementation before conception impairs reversal learning in offspring mice

**DOI:** 10.1038/s41598-017-03158-1

**Published:** 2017-06-08

**Authors:** Kristin S. Henzel, Devon P. Ryan, Susanne Schröder, Marco Weiergräber, Dan Ehninger

**Affiliations:** 1 German Center for Neurodegenerative Diseases (DZNE), Sigmund-Freud-Straße 27, 53127 Bonn, Germany; 2 Research Group Experimental Neuropsychopharmacology, Federal Institute for Drugs and Medical Devices, Kurt-Georg-Kiesinger-Allee 3, 53175 Bonn, Germany; 3 Max Planck Institute for Immunobiology and Epigenetics, Stübeweg 51, 79108 Freiburg, Germany

## Abstract

Maternal folic acid (FA) supplementation prior to and during gestation is recommended for the prevention of neural tube closure defects in the developing embryo. Prior studies, however, suggested that excessive FA supplementation during gestation can be associated with toxic effects on the developing organism. Here, we address whether maternal dietary folic acid supplementation at 40 mg/kg chow (FD), restricted to a period prior to conception, affects neurobehavioural development in the offspring generation. Detailed behavioural analyses showed reversal learning impairments in the Morris water maze in offspring derived from dams exposed to FD prior to conceiving. Furthermore, offspring of FD dams showed minor and transient gene expression differences relative to controls. Our data suggest that temporary exposure of female germ cells to FD is sufficient to cause impaired cognitive flexibility in the subsequent generation.

## Introduction

It has been known for decades that folic acid (FA) supplementation before and during pregnancy reduces the incidence of neural tube defects (NTDs) in newborns^1, 2^. Many countries have therefore established national programs for mandatory FA fortification of food, which has reduced the occurrence of NTDs by up to 46%^3–5^. It has also been recommended that women who might become pregnant should take daily FA supplements to minimise the risk of NTDs^6^. Since the first fortification programs started, global FA intake has increased dramatically. In the United States, for example, this increase was twice as large as anticipated prior to the implementation of fortification programs and, consequently, serum folate concentrations on a population level may be up to 150% higher than in the 1990 s^7, 8^.

While the population-level benefits of FA supplementation are well documented, some previous studies have noted possible adverse consequences of certain FA supplementation regimens. In rat offspring, a change in body weight and metabolism as well as an increased risk for mammary adenocarcinomas are associated with high maternal folate levels^9–11^. In mice, disrupted fetal development, embryonic loss, and embryonic developmental delay can be caused by a high-dose FA supplementation in the maternal food during gestation^12, 13^. In humans, the risk to develop allergies, atopic dermatitis and childhood asthma is increased if the mother consumes large amounts of FA during pregnancy^14–17^. In a previous study in mice we showed that paternal consumption of a methyl donor-rich diet, including elevated concentrations of folic acid, had effects on cognitive and neural functions in offspring mice^18^.

Elevated folate levels have also been linked to epigenetic changes. *In vitro*, gene expression levels shift as a function of the extracellular folate concentration^19^. Maternal folate status in mice has an impact on DNA methylation and gene expression in the offspring’s cerebral hemispheres and at specific loci in the fetal gut^20–22^. High maternal FA also influences gene expression in the cerebellum of mouse pups^23^. Global gene methylation decreases in coagulated blood samples of women after taking daily FA supplements^24^ and children of supplemented mothers show increased *IGF2* methylation^25^.

**Figure 1:**
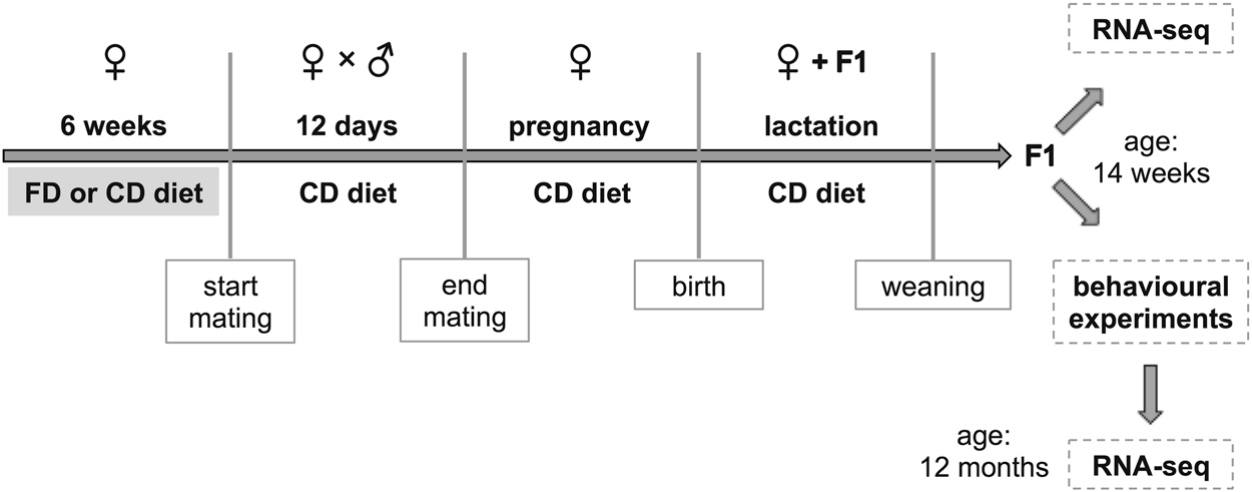
Schematic illustration of the study design.

There are still many unresolved questions concerning possible health consequences of high FA intake. In particular, the impact of maternal FA supplementation on cognitive and behavioural abilities in the offspring remains insufficiently investigated. Moreover, the studies available mostly involve designs with direct exposure of the developing offspring to excessive folate during the entire period of *in utero* development and during early postnatal development. Thus, behavioural and epigenetic effects could be direct consequences of high folate doses upon the developing individual. To address whether dietary FA supplementation prior to conception has effects on the offspring generation, we restricted maternal FA supplementation to a period prior to mating (see Fig. 1 for schematic illustration of study design). Under these conditions, there is exposure of the female, including her germ cells, to high FA doses, while direct gestational effects on the developing organism are limited because excess folate is eliminated rapidly^26–28^. In an earlier study^12^ it was shown that folic acid supplementation at 40 mg/kg diet fed to dams before and during pregnancy caused embryonic defects and developmental delay. We based our FA supplementation regimen on this prior work^12^ and wanted to assess possible effects of this diet on cognition, behaviour and hippocampal gene expression in the offspring of FA-supplemented dams. Our studies revealed clear deficiencies in reversal learning caused by an FA-rich diet fed before mating. As FA supplementation shortly before and during pregnancy has been widely recommended and adopted^29^, these results may provide important insights regarding possible adverse intergenerational effects associated with excessive folate intake that should be studied in more detail in human populations.

## Results

### Spatial learning and reversal learning in the Morris water maze

**Figure 2:**
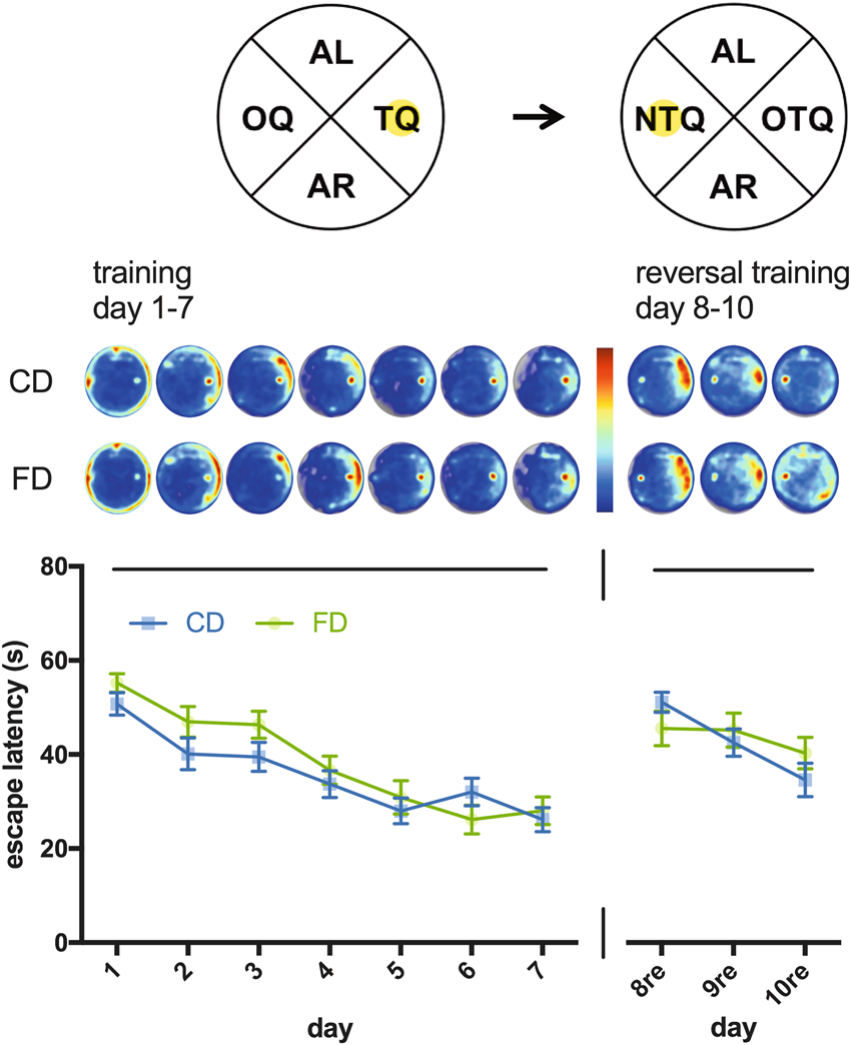
Acquisition and reversal training in the Morris water maze. Visual representations of swim tracks are shown for each day and group. Relative occupancy values during training and reversal training in the Morris water maze are indicated by the colour code (see colour bar; red denotes high occupancy, blue denotes low occupancy values). The yellow circle denotes the platform location during training and reversal training. TQ = target quadrant, AL = adjacent left, AR = adjacent right, OQ = opposite quadrant, OTQ = old target quadrant, NTQ = new target quadrant. CD = F1 offspring of control dams. FD = F1 offspring of dams that received the folic acid-supplemented diet prior to mating. Data are presented as mean ± S.E.M. (CD: *n* = 24, FD: *n* = 20).

We examined spatial learning and memory in CD and FD F1 mice using a hidden version of the Morris water maze. Analysis of escape latencies during the first seven days of training showed comparable acquisition by CD and FD F1 mice (Fig. 2). There was a significant effect of day on escape latencies (two-way ANOVA: F (6, 252) = 23.35, p < 0.0001) but no effect of maternal diet (F (1, 42) = 2.253, p = 0.1408; full statistical analyses of escape latencies can be found in Supplementary Table S1).

**Figure 3:**
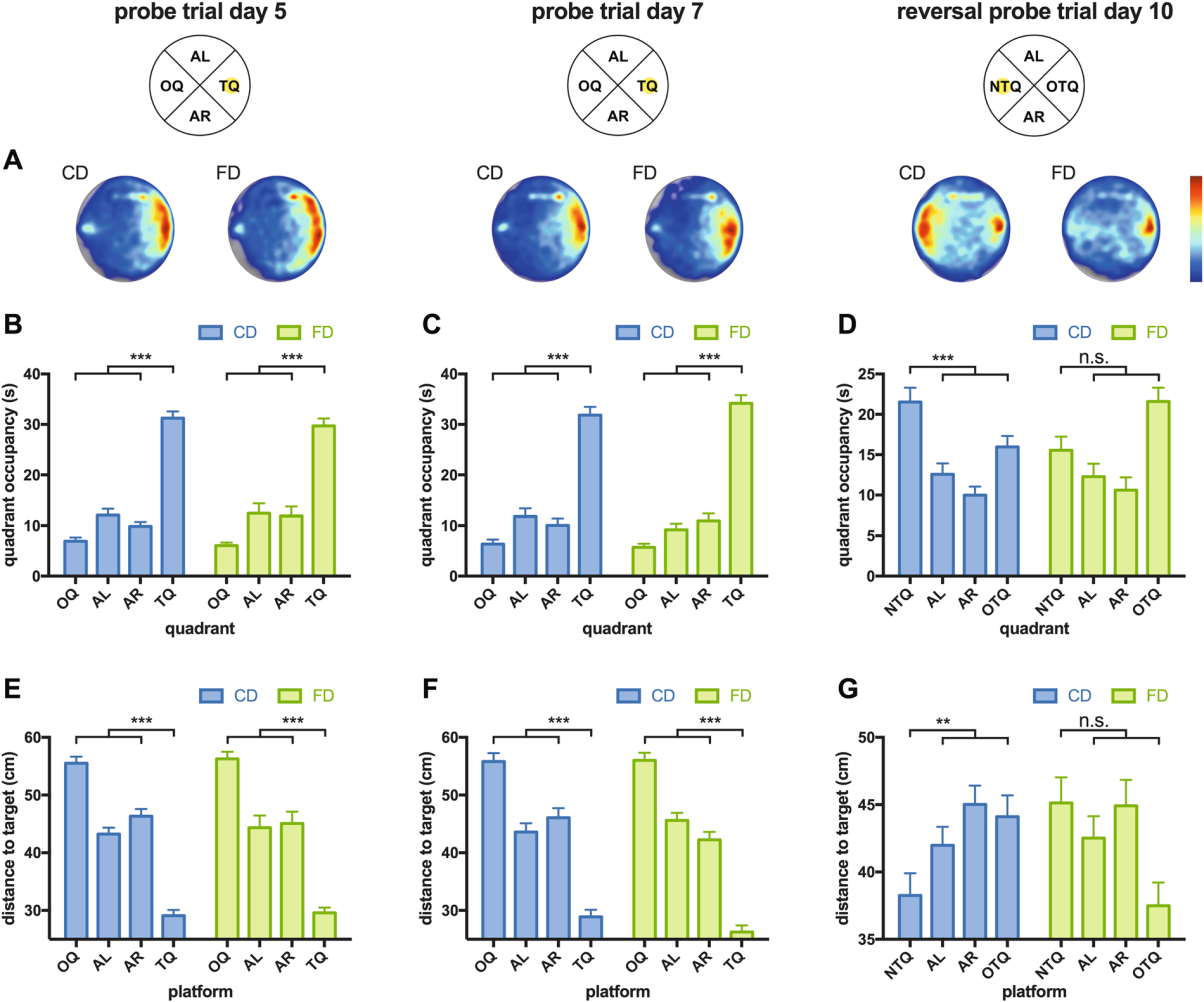
Morris water maze probe trial data. The figure shows Morris water maze probe trial data regarding probe trials delivered on days 5, 7 and 10. (**A**) Cumulative swim track representations (heat maps) are shown for both groups and for each day. Relative occupancy values during the probe trials in the Morris water maze are indicated by the colour code (see colour bar; red denotes high occupancy, blue denotes low occupancy values). (**B–D**) Quadrant occupancy during probe trials on day 5 (**B**), day 7 (**C**) and during the reversal probe trial on day 10 (**D**). (**E–G**) Average distance to the target location (average proximity) during probe trials on day 5 (**E**), day 7 (**F**) and during the reversal probe trial on day 10 (**G**). TQ = target quadrant, AL = adjacent left, AR = adjacent right, OQ = opposite quadrant, OTQ = old target quadrant, NTQ = new target quadrant (former OQ). CD = F1 offspring of control dams. FD = F1 offspring of dams that received the folic acid-supplemented diet prior to mating. Data are presented as mean ± S.E.M; asterisks denote significant differences (**p < 0.01, ***p < 0.001; CD: *n* = 24, FD: *n* = 20).

**Figure 4:**
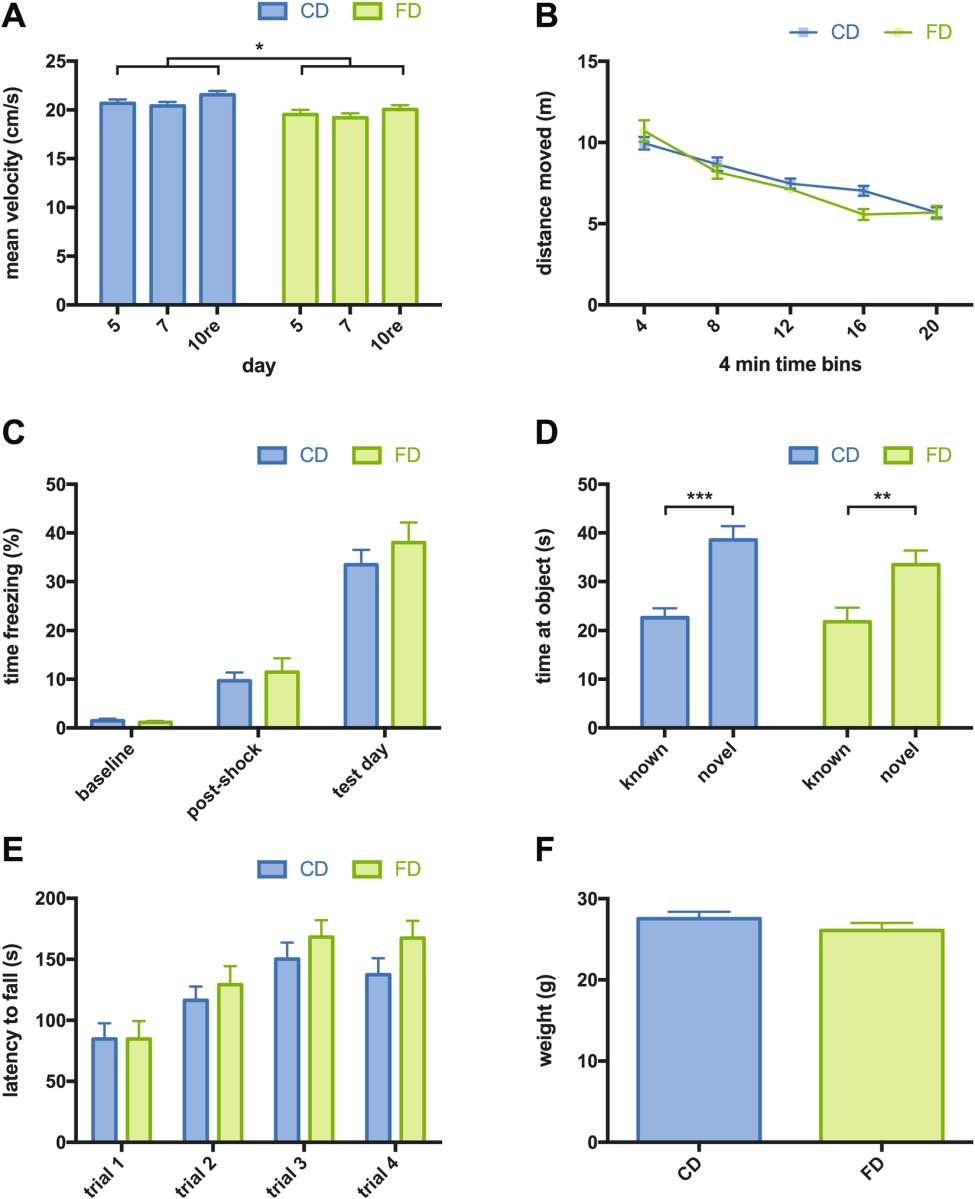
Additional behavioural data and analyses. (**A**) Mean velocity during probe trials in the Morris water maze. (**B**) Distance moved during a 20 min open field test. (**C**) Percentage of time spent freezing during baseline, the post-shock period and during the context test given one day after training in a contextual fear conditioning paradigm. (**D**) Exploration times (known object, novel object) in the context of an object place recognition test. (**E**) Latency to fall off an accelerating rotarod. (**F**) Body weight (at 12 weeks of age). CD = F1 offspring of control dams. FD = F1 offspring of dams that received the folic acid-supplemented diet prior to mating. Data are presented as mean ± S.E.M.; asterisks denote significant differences (*p < 0.05, **p < 0.01, ***p < 0.001; CD: *n* = 24, FD: *n* = 20).

To test how accurately animals had learned the spatial location of the escape platform during training, we performed probe trials, during which the platform was removed from the pool, on days 5 and 7 of water maze training (Fig. 3). Over all probe trials we noted minor differences between CD and FD F1 mice in terms of swim speed (Fig. 4A), which appeared to be slightly reduced in FD offspring (two-way ANOVA: effect of maternal diet: F (1, 42) = 6.878, p = 0.0121). With regards to quadrant occupancy measures, two-way ANOVAs with maternal diet as the between-subjects factor and quadrant as the within-subjects factor revealed significant quadrant effects for probe trials on days 5 and 7 (day 5: F (3, 126) = 97.02, p < 0.0001; day 7: F (3, 126) = 118.9, p < 0.0001). In addition, there was a significant platform effect with regards to proximity measures but no significant quadrant or platform x maternal diet interaction on both probe trials, indicating a similar swim pattern of both groups (for full statistics on probe trials see Supplementary Table S2). Further analyses showed that CD and FD F1 mice spent more time in the target quadrant compared to the average of the other quadrants (t-test: p < 0.0001 for days 5 and 7). Additionally, CD and FD F1 mice showed significantly lower average distances to the position where the target platform was located during training than to equivalent positions in the other quadrants (t-test: p < 0.0001 for days 5 and 7). Target quadrant occupancy and proximity to target did not differ between CD and FD F1 mice on day 5 (t-test: quadrant occupancy: p = 0.4492; proximity: p = 0.7135) and on day 7 (t-test: quadrant occupancy: p = 0.3208; proximity: p = 0.1282). These results indicate that animals of both groups had learned the task and that they had learned it equally well.

Next, we assessed reversal learning in the Morris water maze in CD and FD F1 mice by relocating the escape platform to the centre of the opposite quadrant and providing additional training trials on days 8–10 with the platform in this new location. Comparison of escape latencies during reversal training did not show measurable group differences (two-way ANOVA: effect of maternal diet: F (F (1, 42) = 0.1059, p = 0.7465; Fig. 2). To more closely assess swim patterns we generated combined visual representations of all swim tracks for each group. In the control group, these heat maps showed a strong initial bias during reversal training towards the area of the pool that previously contained the escape platform and this bias disappeared during the course of reversal training. In FD F1 mice, we also observed an initial bias towards the former target quadrant, which appeared to persist, however, to some extent throughout the course of reversal training, raising the possibility that reversal learning was altered in FD F1 mice.

To determine whether this was indeed the case, we delivered a probe trial, during which the platform was removed from the pool, after completion of reversal training (Fig. 3). A two-way ANOVA with maternal diet as the between-subjects factor and quadrant as the within-subjects factor revealed a significant maternal diet × quadrant interaction with regards to both, the quadrant occupancy and proximity measures (quadrant occupancy: F (3, 126) = 3.609, p = 0.0153; proximity: F (3, 126) = 4.599, p = 0.0043), indicating that swim patterns during the reversal probe trial differed as a function of maternal diet. Comparison of the two groups using litter means showed similar results (see Supplementary Fig. S1). Qualitatively similar results were obtained when results from male and female mice were analysed separately (for more information see Supplementary Fig. S2). Additional analyses revealed that control mice displayed significantly higher occupancy of the new target quadrant compared to the average of the other quadrants (t-test: p < 0.0001). Occupancy measures for the old target quadrant did not differ from the average of the other quadrants (t-test: p = 0.5640). In addition, control mice showed lower proximity measures for the new platform position compared to corresponding positions in the other quadrants (t-test: p = 0.004), while proximity measures for the old target location did not differ from the average of the other quadrants (t-test: p = 0.1772). Together, these data clearly show that control mice had learned the new target location after having completed reversal training. Analyses of reversal probe trial data from the offspring of FA-supplemented dams, in contrast, indicated that these animals did not spend more time in the new target quadrant than in the average of the other quadrants (t-test: p = 0.6799), but did spend more time in the quadrant that previously contained the target compared to the average of the other quadrants (t-test: p < 0.0001). Furthermore, FD F1 mice did not show clear differences when proximity values for the new target location were compared with the average proximity of corresponding positions in the other quadrants (t-test: p = 0.0945). FD F1 mice did, however, display significantly lower proximity values for the previous target location compared to the average proximity of corresponding positions in the other quadrants (t-test: p = 0.0014). Swim patterns differing between groups were also evident when heat maps were generated for each group based on the combined visual representations of all reversal probe trial swim tracks. The reversal probe trial demonstrated that, after completion of reversal training, FD F1 mice continued to search in areas close to the previous target location, whereas CD F1 mice showed evidence for more effective learning of the new target location. Collectively, these data show impairments in reversal learning in the Morris water maze in FD F1 mice.

### Locomotor activity in a novel environment

General activity was measured in an open field test and analysed in 4 min time bins (Fig. 4B). With regards to distance moved, a two-way ANOVA with maternal diet as the between-subjects factor and time as the within-subjects factor showed no main effect of maternal diet (F (1, 42) = 0.619, p = 0.4359) but a significant time x maternal diet interaction (F (4, 168) = 3.257, p = 0.0133), indicating that exploratory activity differed as a function of time.

### Context fear conditioning and object place recognition learning

We also examined associative learning in a contextual fear conditioning paradigm in CD and FD F1 offspring. In context fear conditioning (Fig. 4C), freezing times during the context test indicated robust learning in both offspring groups but analysis of freezing times did not reveal apparent group differences for baseline (t-test: p = 0.4772), post-shock (p = 0.5800) or test day measures (p = 0.3783).

CD and FD F1 offspring were also assessed in an object place recognition learning task, during which they initially encountered two identical objects in defined locations of an arena during a training session. For the test session, one of the objects was shifted to a novel location, while the other object remained in the known position within the arena. A two-way ANOVA with maternal diet as the between-subjects factor and object as the within-subjects factor revealed a significant object effect (F (1, 42) = 46.03, p < 0.0001). Both groups spent significantly more time exploring the object in the novel position compared to the object in the known position during the test (t-test: CD: p < 0.0001, FD: p = 0.0066), indicating that they learned and remembered the initial positions of the objects (Fig. 4D). There was no significant effect of maternal diet (two-way ANOVA: F (1, 42) = 0.8984, p = 0.3486). The results of these two additional learning tests show, in line with the water maze data, that initial learning was not disrupted in FD F1 offspring.

### Motor coordination and balance

In order to assess motor coordination and balance, we measured latencies to fall off an accelerating rotarod in CD and FD F1 offspring. Repeated-measures two-way ANOVA with maternal diet as the between-subjects factor and trial as the within-subjects factor revealed a significant main effect of trial (F (3, 126) = 48.86, p < 0.0001) but no significant effect of maternal diet (F (1, 42) = 0.7746, p = 0.3838) on time spent on the accelerating rod (Fig. 4E). There was no discernable difference in body weight between CD and FD F1 groups (t-test: p = 0.2514; Fig. 4F). Accordingly, maternal diet had no influence on gross motor coordination in FD F1 offspring.

### Hippocampal gene expression

**Figure 5:**
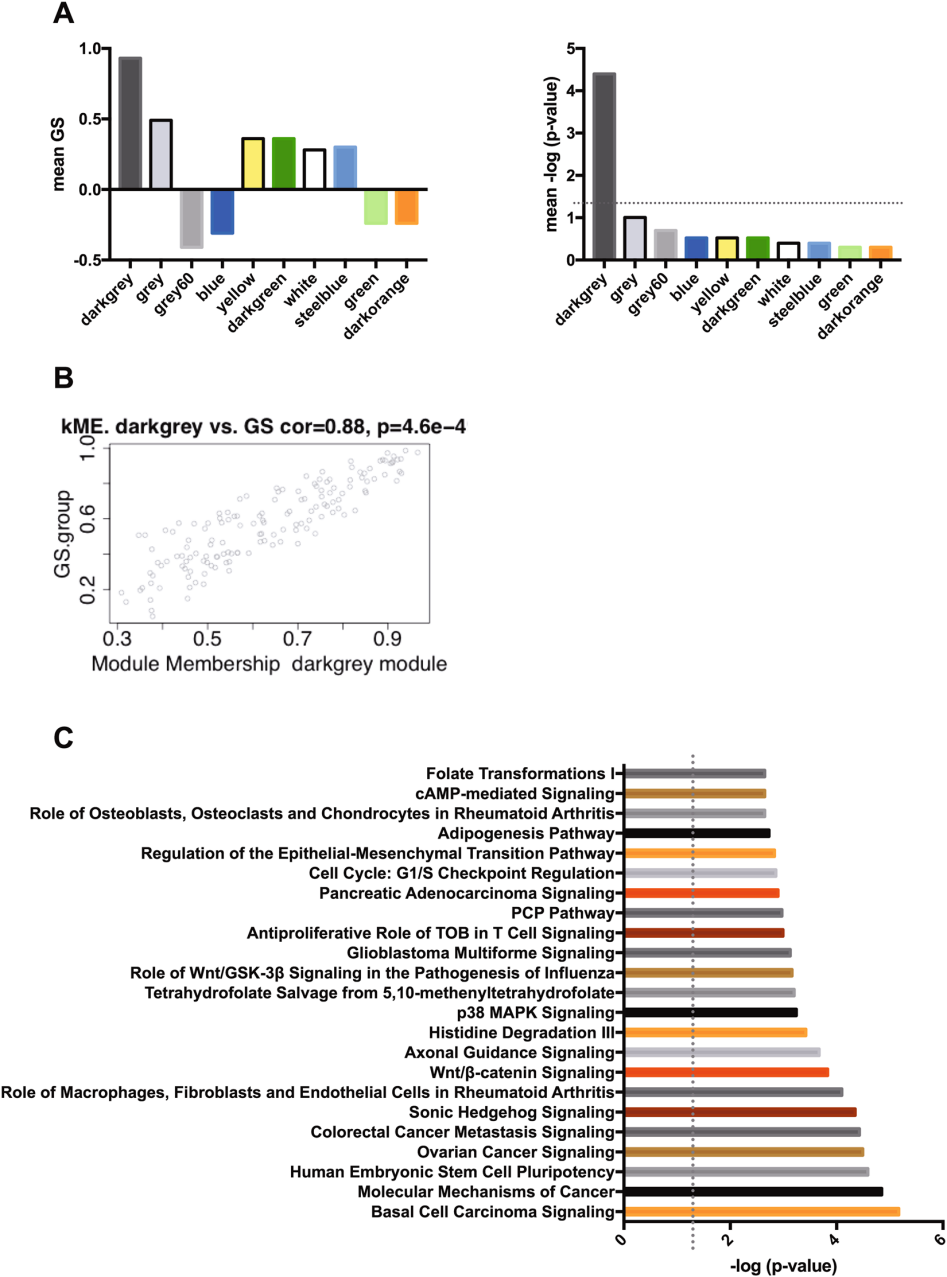
Weighted correlation network analysis (WGCNA) of hippocampal expression data in CD and FD F1 mice. (**A**) Graphs show module-trait relationships and corresponding p-values (presented as –log of the p-value) of the top ten modules detected via WGCNA (module names are provided on the x-axes). The p-value threshold of –log (0.05) is indicated by a dotted line. (**B**) Scatterplot of gene significance (GS.group) versus module membership (kME) for the darkgrey module. We observed a strong correlation between GS values for group (maternal diet) and module membership. (**C**) The graph shows the top enriched canonical pathways among genes with membership in the darkgrey module.

**Figure 6:**
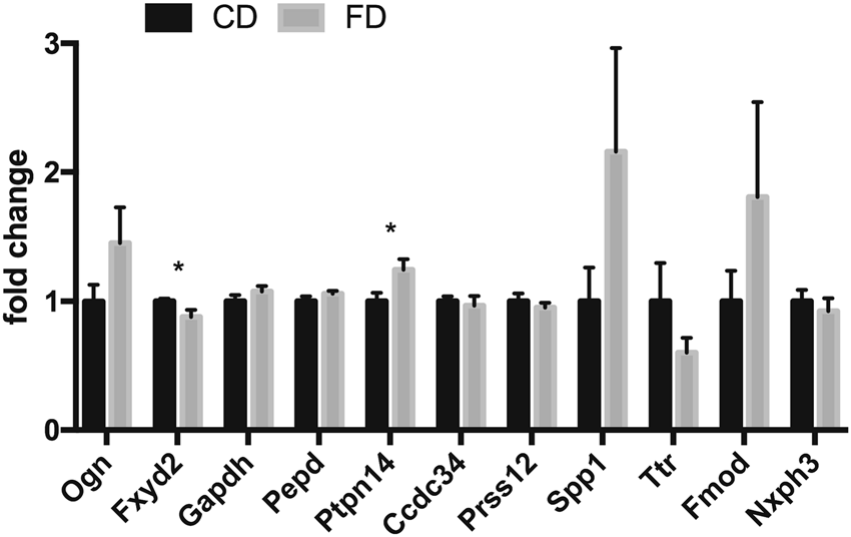
Expression changes of candidate genes in hippocampi of CD and FD F1 mice evaluated by qPCR. The graph shows normalized fold change values relative to control samples. Data are presented as mean ± S.E.M.; asterisks denote significant differences (*p < 0.05; *n* = 6 per group).

Hippocampal RNA of CD and FD offspring (either 14 weeks or 12 months of age) was extracted and subjected to RNA-seq. Results of the RNA-seq-based differential expression analysis of the 14-weeks-old cohort are summarized in the supplemental material. The top 15 genes with an adjusted p-value < 0.1 are shown in Table 1. We further analysed the data performing a weighted correlation network analysis (WGCNA), which is a powerful tool to identify gene networks, to find and summarize clusters (modules) and to relate those modules to external traits like the group membership (CD or FD F1). WGCNA identified one gene co-expression module (“darkgrey module”) significantly correlated with maternal diet (R^2^ = 0.93, p < 0.001; Fig. 5A,B). Pathway analysis of genes within the darkgrey module yielded a significant enrichment of a number of pathways among those genes (Fig. 5C), including pathways related to FA metabolism and Wnt/β-catenin signalling. Additional qPCR analyses of selected genes showed a significant downregulation of Na^+^/K^+^-ATPase subunit gamma (*Fxyd2*, one-tailed t-test: p = 0.035) and a significant upregulation of Tyrosine-protein phosphatase non-receptor type 14 (*Ptpn14*, one-tailed t-test: p = 0.023) in hippocampi of FD offspring relative to CD offspring (Fig. 6). The direction of changes was identical to the results obtained by RNA-seq. Comparison of global hippocampal gene expression in a cohort of older CD and FD F1 mice (12 months old) showed no apparent group differences (data not shown).

**Table 1:** Differential expression analysis of hippocampal gene expression data from CD and FD F1 mice.

Ensembl ID	gene name	log_2_ fold change	p-value	adjusted p-value
ENSMUSG00000083863	predicted gene 13341	0.488044	0.000000	0.000000
ENSMUSG00000081896	predicted gene 5389	0.353805	0.000000	0.000000
ENSMUSG00000084289	predicted gene 6977	0.317269	0.000000	0.000404
ENSMUSG00000057990	RIKEN cDNA E030024N20 gene	0.140377	0.000002	0.008272
ENSMUSG00000083391	predicted gene 14148	0.150488	0.000004	0.015272
ENSMUSG00000081164	predicted gene 8722	0.132908	0.000007	0.022477
ENSMUSG00000063129	aldolase 1 A, retrogene 2	0.196374	0.000008	0.022477
ENSMUSG00000069236	predicted pseudogene 7251	0.162689	0.000009	0.022477
ENSMUSG00000078592	predicted gene 4609	0.095478	0.000014	0.033356
ENSMUSG00000094852	predicted pseudogene 9034	0.130803	0.000025	0.047908
ENSMUSG00000083219	predicted gene 11410	0.158353	0.000026	0.047908
ENSMUSG00000097480	predicted gene 9499	0.108404	0.000027	0.047908
ENSMUSG00000082454	predicted gene 12183	0.226359	0.000053	0.086401
ENSMUSG00000081920	predicted gene 15359	0.100836	0.000067	0.095912
ENSMUSG00000082185	predicted gene 12428	0.163640	0.000068	0.095912

The table shows the top of the list of a differential expression analysis of hippocampal RNA sequencing data (adjusted p-value < 0.1).

## Discussion

It has been long known that sufficient maternal folate intake is important for the proper closure of the neural tube during an embryo’s development. Case-control studies have shown that FA supplementation has significantly reduced the incidence of neural tube defects in humans^30–32^. If no FA supplements are taken in early pregnancy there is also a higher risk for children to present with emotional and behavioural problems at the age of 18 months^33^. Nevertheless, to our knowledge, it remained untested whether high-dose dietary FA intake consumed prior to conception may have adverse effects on neurological development in the offspring. In this study, we therefore investigated, in a mouse model, the effects of an FA-rich maternal diet fed only before pregnancy and found notable cognitive impairments in reversal learning in the F1 generation.

In humans, the standard recommendation for daily dietary folic acid intake is 300–400 µg for women^6, 34^. Folate levels in human populations, however, have increased considerably since the first fortification programs started. For example, in Canada 63.5% of the population have a red blood cell folate concentration >1090 nmol/L indicating an intake of >2.1 mg dietary folate equivalent per day^35^. For high-risk pregnancies, some studies even recommend supplementary FA intake of 5 mg/day, which is almost 17x higher than the standard recommendation for women^36^. The amount of FA in our supplemented diet (40 mg/kg chow) therefore represents those high intake rates as it is about 17.4x higher than in our control diet (2.3 mg/kg chow, which meets the standard recommendations for FA in mouse diets^37^). Accordingly, our experiment represented an initial effort to test the consequences of high-dose dietary FA supplementation restricted to a period prior to conception.

During our behavioural experiments, we observed reduced reversal learning abilities in a Morris water maze task in the F1 offspring of FA-supplemented dams. Spatial memory impairments have also been detected in one study where rats were exposed directly to an FA-rich diet for 30 days^38^. Effects on offspring, however, were not investigated in that study. Deficits in reversal learning during the Morris water maze task can be attributed to a form of cognitive flexibility, i.e. the ability to change a previously acquired behaviour and to solve a new spatial task. The inflexibility to change in routine may also be viewed as a form a perseverative behaviour and assessments of reversal learning are, therefore, used as a behavioural endpoint in studies of rodent models for autism spectrum disorders (ASD)^39^. Indeed, available human data indicate a possible increase in offspring ASD risk if maternal folate levels exceed recommendations^40^.

In addition to the effects on reversal learning, we observed a slightly decreased swim speed in offspring of high folate dams during Morris water maze testing. This outcome is likely not based on disturbed motor skills, as the performance of FD F1 mice on an accelerating rotarod was comparable to the performance of CD F1 mice. The differences in velocity could rather indicate a slightly decreased motivation in offspring of high folate dams. A deficit in motivation was also detected in rats directly fed a FA-rich diet^38^.

Another study in mice described increased anxiety and gender-specific hyperactivity due to FA supplementation^20^. We did not observe increased locomotor activity in the open field assay in FD offspring. However, this prior study^20^ provided the FA-rich diet during the entire course of gestation, lactation and even after weaning, which means that the offspring mice that were tested were on the same diet as their mothers. Additionally, FA concentrations differed substantially between the two studies.

In our study design, we restricted the FA-rich diet to a period prior to mating, suggesting that the behavioural alterations in F1 offspring may be a consequence of FA effects on female germ cells, such as epigenetic changes in oocytes induced by excess FA. Since the hippocampus is important for spatial learning and navigation, we tested for altered hippocampal gene expression in offspring mice. We detected only minor alterations in expression profiles between CD and FD offspring. At 14 weeks of age, weighted correlation network analysis combined with enrichment analyses showed a possible connection of the maternal FA-rich diet to alterations in the Wnt/β-catenin signalling pathway in FD offspring mice. Available evidence indicates that canonical Wnt signalling is sensitive to the amount of dietary FA (for review see ref. 41). In addition, our data revealed a downregulation of *Fxyd2* and an upregulation of *Ptpn14* in FD F1 mice. Fxyd2 is a regulator of Na^+^/K^+^-ATPase activity and, thus, important for neuronal excitability and neurotransmission^42^. In rats, high-dose maternal folic acid supplementation affected offspring synaptic transmission and decreased offspring seizure threshold^43^. Therefore, decreased expression of the gamma subunit of Na^+^/K^+^-ATPase in offspring of dams fed a FA-rich diet before mating could potentially contribute to altered neural functions and cognitive abilities in these mice.

By the age of 12 months, global gene expression differences between CD and FD F1 mice were no longer detectable. One likely scenario could be that gene expression differences diminish with age. In cerebral hemispheres of pups whose mothers were fed a FA-rich diet throughout gestation, changes in methylation and gene expression have previously been observed at postnatal day 1^20, 22^. It is conceivable that changes due to a diet fed before mating are only detectable early in life and further studies are needed to clarify this aspect.

In principle, it is also possible that the behavioural phenotype in FD offspring that we observed is due to a diet-induced change in the behaviour of the dam. Variations in nursing behaviour, for example, could also lead to an alteration in behaviour of the offspring. To investigate that possibility, follow-up studies will need to examine the nursing behaviour of the dam or use IVF-mediated approaches with foster dams to exclude direct contact between mothers and offspring. A limitation of the present study is that cognitive flexibility was only assessed in the Morris water maze. It will be of interest to investigate reversal learning in offspring of FA-supplemented dams in additional behavioural tasks using, for example, Y-maze-based reversal learning in future studies.

In conclusion, we found alterations in the cognitive phenotype of offspring of dams fed a diet with high FA concentrations before mating. Our results complement existing studies that document adverse effects of excessive FA intake when applied throughout gestation^12, 43^. Given that FA intake in human populations increased considerably since the first fortification programs started^3, 7, 44^, it should be of public interest to ensure that population FA intake is kept within an optimal range between FA deficiency and FA excess.

## Material and Methods

### Animals

All mice were housed under SPF conditions with 2–5 mice per cage on a 12-h light/dark cycle with food and water available *ad libitum*. Female young adult 129S6/SvEv mice were purchased from Taconic (Denmark). After an acclimation phase of several weeks, the mice were randomly assigned to two groups. One of the groups (CD) received an AIN-93G control diet with the standard amount of 2.3 mg FA/kg diet (Altromin, Germany). The FA-supplemented group (FD) was fed a modified version, which contained 40 mg FA/kg diet (Altromin, Germany) and was otherwise identical to the control diet. After 6 weeks of feeding, females were individually mated with young adult C57BL/6 J males purchased from Charles River (Germany). From mating onwards, all mice received the control diet with FA at 2.3 mg/kg diet. Thus, the FA-rich diet was only fed before mating and not during gestation or weaning (Fig. 1). After 12 days, the males were removed from the cages.

All experiments were performed in accordance with the German Animal Health and Welfare Act (German Federal Law, §8 Abs. 1 TierSchG). The present study was approved by “Landesamt für Natur, Umwelt und Verbraucherschutz Nordrhein-Westfalen” (Recklinghausen, Germany).

### Behavioural experiments

Experiments were carried out with adult 129S6/SvEv x C57BL/6 J F1 offspring of supplemented or non-supplemented dams with *n* = 20 FD F1 mice and *n* = 24 CD F1 mice. We chose this genetic background because 129S6/SvEv x C57BL/6J F1 hybrids are isogenic and they show superior performance on learning and memory tasks^45^. Mice used were females and males originating from at least 6 different dams and litters per group with 2–6 mice coming from the same litter. Before starting the experiment, all mice were handled for 5 days for approximately 2 min/day/animal. Behavioural assessments were started at the age of 14 weeks. We carried out behavioural studies during the light phase of the day. All experiments were conducted blind to treatment condition.

### Morris water maze

The Morris water maze was performed as previously described^46, 47^ with the following modifications. Mice received two practice trials per day for 7 days with probe trials on day 5 and day 7. Each mouse had to learn to find a hidden platform under the water surface of a pool filled with opaque water. The starting position was randomly altered for every trial. To assess how accurately animals had learned the platform position during training, we analysed the time the mice spent in the target quadrant during the probe trials and compared this to the time spent in the other quadrants (quadrant occupancy). Furthermore, we analysed, for probe trials, the average distance the mouse kept from the position that contained the platform during training and compared this to the distance to equivalent positions in the other three quadrants (proximity).

For reversal learning, the hidden platform was placed in the centre of the opposite quadrant of the pool (i.e., the one opposite to the previous target quadrant) and mice were trained for 3 more days (2 trials per day) to learn the new location of the platform. On day 10, an additional probe trial was performed (during which the platform was removed from the pool). The time the mouse spent searching for the platform in the new target quadrant was measured and compared to the time spent searching in the other quadrants. We also analysed velocity and distance to the target position during probe trials as well as escape latencies during all training trials via an automated system (Ethovision XT, Noldus). Cumulative heat maps for each group were created for all training days and probe trials in Ethovision XT (Noldus).

### Open field

The general exploratory behaviour of mice was tested in an open field task as previously described^48^ with the modifications described below. Each mouse was placed individually in an acrylic box (27 cm × 27 cm) for 20 min and behaviour was recorded for offline analysis. Light intensity during the test was set to approx. 150 lux in the centre of the test arena. Activity (total distance moved) was measured and analysed in 4 min time bins by an automated system (Ethovision XT, Noldus).

### Context fear conditioning

To assess conditioned fear, we used a video fear conditioning system (Med Associates) essentially as previously described^49^. Mice received a single training session of 181 s with one 0.75 mA shock (1 s) after 120 s. On the next day, animals were subjected to a 180 s context test.

### Object place recognition

This test was carried out in acrylic boxes (27 cm × 27 cm) with non-transparent white walls containing a visible cue (small, green triangle) in the centre of one of the walls. The experiment was performed on 5 consecutive days. On day 1, each mouse was placed in the empty arena for 20 min to habituate the animal to the setting. On days 2–4, identical conical flasks were placed in two specific positions of the arena and the animals were allowed to explore for 20 min each. On day 5, one of the objects was moved to a new position and the behaviour of the mouse was recorded for 6 min. Exploration times of objects during the test were quantified by an experienced experimenter blind to treatment group and object location.

### Rotarod

Motor skills of the mice were examined using an accelerating rotarod (Med Associates). Each mouse was placed on an accelerating rod (4–40 rpm) for a maximum of 5 min. An automated electric light beam (Med Associates) was used to measure the time until the mouse fell off the rod. The trial was stopped manually if the mouse clung to the rod for two consecutive rotations without actually running on the rod (passive cycling). The latency to fall off the rotating rod was compared between groups. Animals were given 2 daily training trials for 2 consecutive days.

### Statistical analysis

Statistical analyses were conducted using R^50^ or GraphPad Prism Version 6.0e. Analyses of behavioural experiments were performed using two-way analysis of variance (ANOVA) or two-tailed, unpaired Student’s t-test unless stated otherwise. A p-value less than 0.05 was considered to be statistically significant. All statistical tests are described in detail in the main text.

### Tissue preparation and RNA extraction

Mice were killed via cervical dislocation. After hippocampal extraction, tissue was flash-frozen in liquid nitrogen and stored at -80 °C. RNA was extracted from whole hippocampus using the RNeasy Mini Kit (Qiagen) according to the manufacturer’s instructions. RNA concentrations were measured via NanoDrop (Thermo Scientific).

### RNA sequencing

For RNA sequencing (RNA-seq) we first validated that hippocampi were extracted without any adjacent tissues, especially choroid plexus. Towards this end, we employed qPCR to assess the extracted RNA for the presence of choroid-specific transcripts. cDNA was prepared using the iScript^TM^ cDNA Synthesis Kit (Bio-Rad) and gene expression of orthodenticle homolog 2 (*Otx2*) and prolactin receptor (*Prlr*) was measured using TaqMan® Gene Expression Assays and StepOnePlus™ Real-Time PCR Systems (Applied Biosystems). Almost no expression of *Otx2* and *Prlr* should be detectable in RNA extracted from pure hippocampus. *Actb* was used as an endogenous control.

In preparation of RNA-seq analyses, the quality and quantity of input RNAs were determined with a 2100 Bioanalyzer (Agilent Technologies) using the RNA 6000 Nano Kit. Input RNAs were used to create a next generation sequencing library (Illumina). All libraries were run on an Illumina HiSeq2000 (50 bp single end sequencing). We sequenced RNA from a younger (i.e., 14 weeks old) cohort of F1 offspring mice (*n* = 6 mice of the CD F1 group and *n* = 5 mice of the FD F1 group) and, in a separate experiment, from an older (i.e., 12 months old) cohort of animals (*n* = 6 mice per group). Reads were quality and adapter trimmed with custom software. The resulting trimmed reads were aligned against the mouse mm10 genome with STAR 2.4.0^51^. Unique alignments per gene were counted with featureCounts^52^ and the results imported into R^50^ for analysis with DESeq2^53^. An adjusted p-value threshold of 0.1 was used to determine significance.

For the differential gene expression data from the younger cohort, Weighted Correlation Network Analysis (WGCNA) in R was used in order to identify modules that were correlated with maternal diet. Analysis was basically performed as described elsewhere^54, 55^. Ingenuity Pathway Analysis (IPA; Ingenuity Systems Inc., Redwood, CA, USA) was used for integrated analysis of altered gene expression.

The RNA-seq data of the 14-week-old cohort have been deposited in NCBI’s Gene Expression Omnibus^56^ and are accessible through GEO Series accession number GSE80587.

### qPCR

For validation and deeper analysis of the RNA-seq results 11 candidate genes with mean expression levels >50 and a p-value < 0.1 were additionally assessed via qPCR. The iScript^TM^ cDNA Synthesis Kit (Bio-Rad) was used to reverse transcribe extracted RNA into cDNA and qPCR was performed with commercially available TaqMan® Gene Expression Assays on a StepOnePlus™ Real-Time PCR System (Applied Biosystems). Beta-2-microglobulin (*B2m*) was used as endogenous control and gene expression in hippocampi of FD F1 mice was normalized and analysed relative to that measured in CD F1 mice.

## Electronic supplementary material

Supplementary information

Dataset 1
