# Supplementary material for: High-dose maternal folic acid supplementation before conception impairs reversal learning in offspring mice
Source: Sci Rep. 2017 Jun 8;7:3098. doi: 10.1038/s41598-017-03158-1 (PMC5465191; doi:10.1038/s41598-017-03158-1)
Supplement: Supplementary file 1 — Supplementary information [file 41598_2017_3158_MOESM1_ESM.pdf]

High-dose maternal folic acid supplementation before conception impairs reversal learning in offspring mice

Kristin S. Henzel[1], Devon P. Ryan[1,*], Susanne Schröder[1], Marco Weiergräber[2] and Dan Ehninger[1]

[1]German Center for Neurodegenerative Diseases (DZNE), Sigmund-Freud-Straße 27, 53127 Bonn, Germany;

[2]Department of Neuropsychopharmacology, Federal Institute for Drugs and Medical Devices, Kurt-Georg-Kiesinger-Allee 3, 53175 Bonn, Germany;

[*]Present address: Max Planck Institute for Immunobiology and Epigenetics, Stübeweg 51, 79108 Freiburg, Germany

Correspondence to Dr. Dan Ehninger: Dan.Ehninger@dzne.de

Supplementary Information

Supplementary Tables

Supplementary Table S1: Statistical analyses of escape latencies during training and reversal training in the Morris water maze task. The table shows results of two-way ANOVAs with maternal diet as the between-subjects factor and day as the within-subjects factor.

Measure	Trial type	Day	ANOVA table	F (DFn, Dfd)	p-value
Escape latency	Training	1-7	Interaction<br>Day<br>Maternal diet	F (6, 252) = 1.170<br>F (6, 252) = 23.35<br>F (1, 42) = 2.253	0.3227<br>< 0.0001<br>0.1408
Escape latency	Reversal training	8-10	Interaction<br>Day<br>Maternal diet	F (2, 84) = 1.777<br>F (2, 84) = 6.278<br>F (1, 42) = 0.1059	0.1754<br>0.0029<br>0.7465

Supplementary Table S2: Statistical analyses of probe trial data regarding the trials given on days 5 and 7, as well as the reversal probe trial delivered on day 10 of the Morris water maze task. The table shows results of two-way ANOVAs with maternal diet as the between-subjects factor and quadrant or platform as the within-subjects factor.

Measure	Trial type	Day	ANOVA table	F (DFn, Dfd)	p-value
Quadrant occupancy	Probe	5	Interaction<br>Quadrant<br>Maternal diet	F (3, 126) = 0.5249<br>F (3, 126) = 97.02<br>F (1, 42) = 0.0	0.6660<br>< 0.0001<br>> 0.9999
Quadrant occupancy	Probe	7	Interaction<br>Quadrant<br>Maternal diet	F (3, 126) = 0.8986<br>F (3, 126) = 118.9<br>F (1, 42) = -12.60	0.4440<br>< 0.0001<br>> 0.9999
Quadrant occupancy	Reversal probe	10	Interaction<br>Quadrant<br>Maternal diet	F (3, 126) = 3.609<br>F (3, 126) = 11.88<br>F (1, 42) = 14.00	0.0153<br>< 0.0001<br>0.0005
Proximity	Probe	5	Interaction<br>Platform<br>Maternal diet	F (3, 126) = 0.2473<br>F (3, 126) = 108.5<br>F (1, 42) = 0.1654	0.8631<br>< 0.0001<br>0.9991
Proximity	Probe	7	Interaction<br>Platform<br>Maternal diet	F (3, 126) = 1.627<br>F (3, 126) = 125.8<br>F (1, 42) = 1.507	0.1864<br>< 0.0001<br>0.2265
Proximity	Reversal probe	10	Interaction<br>Platform<br>Maternal diet	F (3, 126) = 4.599<br>F (3, 126) = 1.953<br>F (1, 42) = 0.06285	0.0043<br>0.1245<br>0.8033

Supplementary Figures

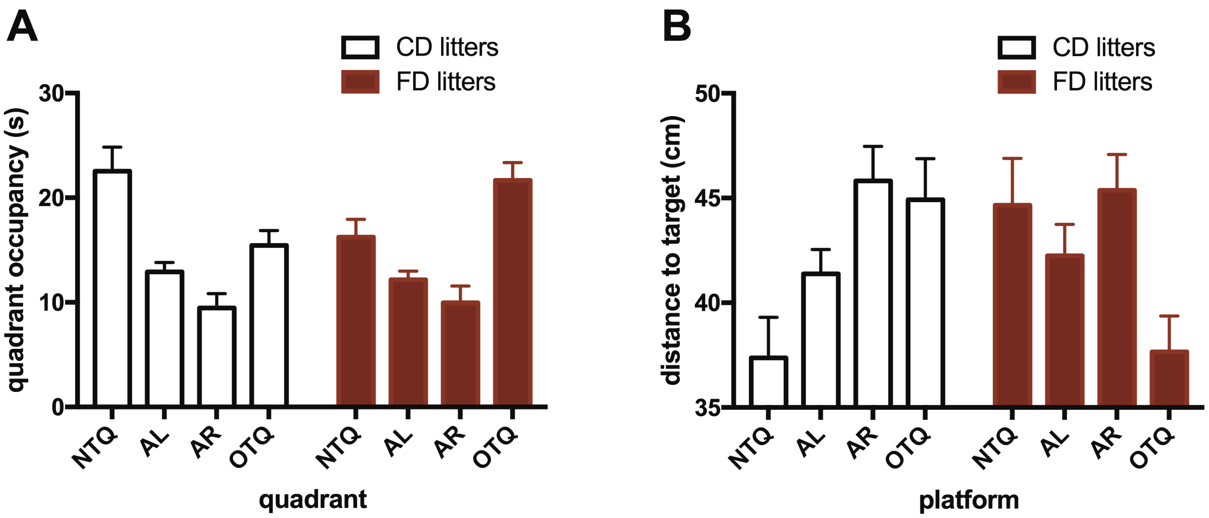

Supplementary Figure S1: Average litter performance during the reversal probe trial on day 10 of the Morris water maze task. A) Quadrant occupancy values and B) average distance to target location of litters of dams fed a FA-supplemented diet before mating (FD) or control dams (CD). According to a 2-way ANOVA with maternal diet as the between-subjects factor and quadrant or platform position as the within-subjects factor there was a significant maternal diet x quadrant interaction (F (3, 30) = 4.177, p = 0.0139) as well as a significant maternal diet x platform interaction (F (3, 30) = 4.993, p = 0.0063). AL = adjacent left, AR = adjacent right, OTQ = old target quadrant, NTQ = new target quadrant. Data are presented as mean ± S.E.M (CD litters: *n* = 6, FD litters: *n* = 6).

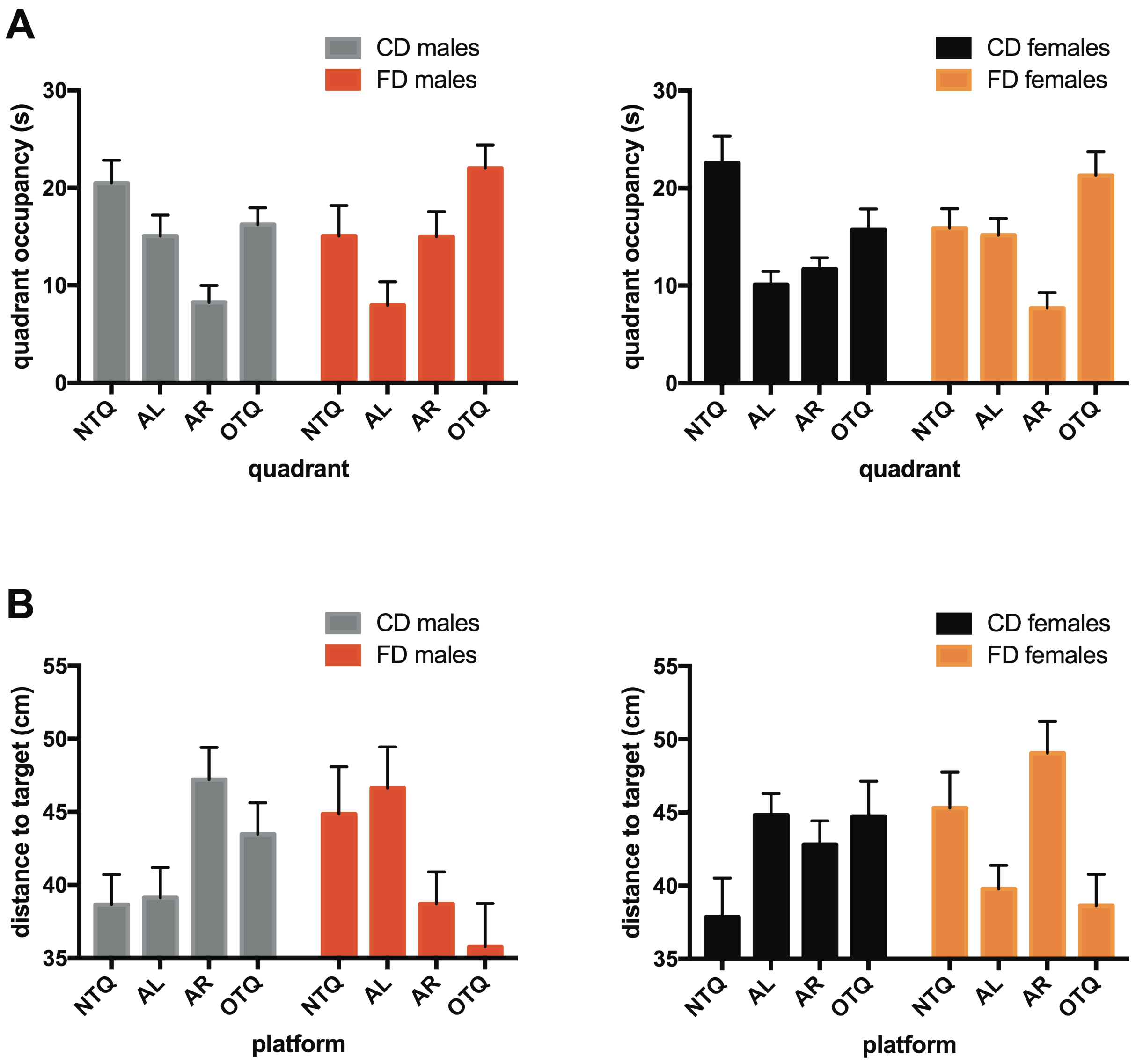

Supplementary Figure S2: Reversal probe trial performance of males and females on day 10 of the Morris water maze task. A) Quadrant occupancy values and B) average distance to the target location of offspring of dams fed a FA-supplemented diet before mating (FD) or offspring of control dams (CD). Analysis was performed using 2-way ANOVA with maternal diet as the between-subjects factor and quadrant or platform position as the within-subjects factor. For both, males and females, there was a significant maternal diet x quadrant interaction (males: $F(3, 54) = 3.698$, $p = 0.0171$; females: $F(3, 66) = 3.757$, $p = 0.0149$) and a significant maternal diet x platform interaction (males: $F(3, 54) = 4.995$, $p = 0.0039$; females: $F(3, 66) = 4.954$, $p = 0.0037$). AL = adjacent left, AR = adjacent right, OTQ = old target quadrant, NTQ = new target quadrant. Data are presented as mean ± S.E.M (CD females: $n = 12$, CD males: $n = 12$, FD females: $n = 12$, FD males: $n = 8$).
